# Bilateral interstitial keratitis following COVID-19: a case report

**DOI:** 10.1186/s12886-023-03160-4

**Published:** 2023-10-13

**Authors:** Nathalie Dalloul Daher, Zeba A. Syed

**Affiliations:** Cornea Service, Wills Eye Hospital, Sidney Kimmel Medical College at Thomas Jefferson University, 840 Walnut Street, Suite 920, Philadelphia, PA 19107 USA

**Keywords:** Interstitial keratitis, Severe acute respiratory syndrome coronavirus 2 (SARS-CoV-2), COVID-19

## Abstract

**Background:**

Although the primary target of severe acute respiratory syndrome coronavirus 2 is the respiratory tract, the expression of the angiotensin-converting enzyme 2 receptor in other tissues facilitates viral entry in others parts of the body, including ocular structures. Ocular manifestations may occur before, during, or after systemic infection.

**Case presentation:**

We report the case of a 60-year-old male who presented with bilateral interstitial keratitis after the onset of COVID-19, with ocular symptoms starting within 7 days after systemic symptoms. Laboratory investigation did not identify any alternative etiology for his disease, although the possibility of Epstein-Barr virus or herpes simpex virus could not be definitively ruled out. The patient had already developed significant corneal scarring and visual debilitation by the time topical steroids were initiated, and his final corrected visual acuity with rigid gas permeable contact lenses was 20/50 and 20/80 in the right and left eye, respectively.

**Conclusions:**

The involvement of ocular tissue by the virus can lead to permanent sequelae such as severe visual loss, and clinicians should be aware of and recognize ophthalmic manifestations of this disease to prompt early intervention.

## Background

Since initial reports of the severe acute respiratory syndrome coronavirus 2 (SARS-CoV-2) in 2019 [1, 2], COVID-19 has affected millions in a worldwide pandemic [1]. This virus uses the angiotensin-converting enzyme 2 (ACE2) receptor to enter host cells in various organs, including ocular structures [3].

Different forms of ocular involvement have been reported including conjunctivitis [1], episcleritis [4], and acute corneal graft rejection [5]. Ocular manifestations may occur before, during, or after systemic infection, and there is no evidence that ocular involvement is related to the severity of the systemic disease [6]. Direct inoculation of the virus in the conjunctiva, transmission via the nasolacrimal duct, and dissemination through conjunctival vessels are mechanisms through which SARS-CoV-2 can infect the ocular surface [7]. We present a rare case of bilateral interstitial keratitis (IK) in a 60-year-old man shortly after the onset of COVID-19 systemic disease. Bilateral IK related to COVID-19 has been previously reported [8]; the mechanism may involve inflammation secondary to SARS-CoV-2 particles, or reactivation or coinfection by other pathogens including Epstein-Barr virus or herpes simpex virus.

### Case presentation

A 60-year-old man was referred for evaluation of bilateral blurry vision, worse in the left eye. He reported no medical history and was not on any systemic medications. Ocular history was notable for allergic conjunctivitis and bilateral primary open-angle glaucoma, and intraocular pressures were successfully controlled with dorzolamide-timolol 22.3/6.8 mg/mL twice daily in both eyes and bimatoprost 0.01% nightly in both eyes. He occasionally used preservative-free artificial tears when allergy symptoms flared. The patient had documented normal corneal evaluations prior to symptom onset.

The patient was diagnosed with COVID-19 three months prior to presentation, confirmed by polymerase chain reaction (PCR) testing of a nasal swab specimen. Systemic symptoms lasted for approximately 7 days and included sore throat, chills, and nasal congestion. The patient experienced mild redness, pain, photophobia, and decreased vision in both eyes, left worse than right, towards the end of his systemic syndrome. He attributed his ocular symptoms to allergies and did not seek medical attention. However, his ocular symptoms significantly worsened after his systemic symptoms resolved. One month later, the patient was evaluated by his primary ophthalmologist and found to have bilateral interstitial keratitis. He was treated for presumed herpetic infection with oral valacyclovir 1 g three times daily for 10 days and topical prednisolone acetate 1% four times daily in both eyes. Symptoms improved over the ensuing 2 weeks, however, because bilateral visual acuity remained compromised, the patient was referred to our institution for corneal evaluation.

**Fig.1:**
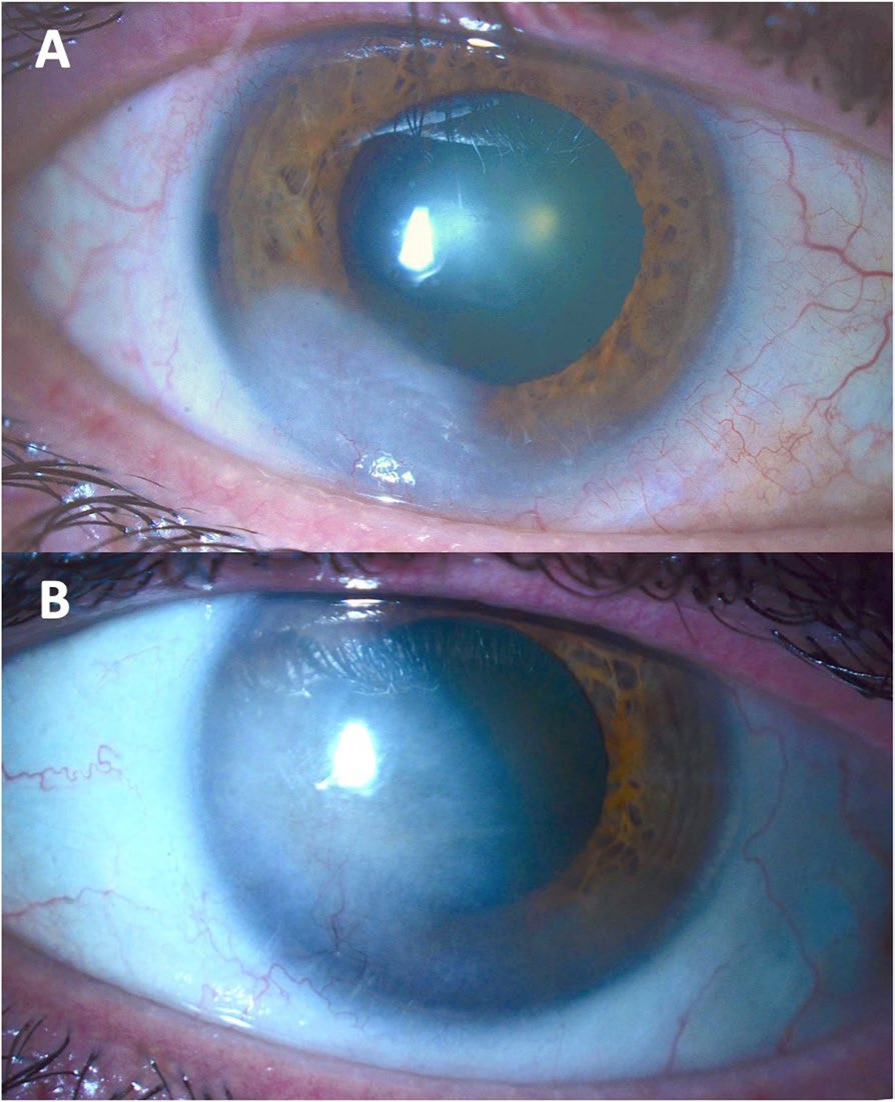
External photography of the right (A) and left (B) eye

**Fig. 2:**
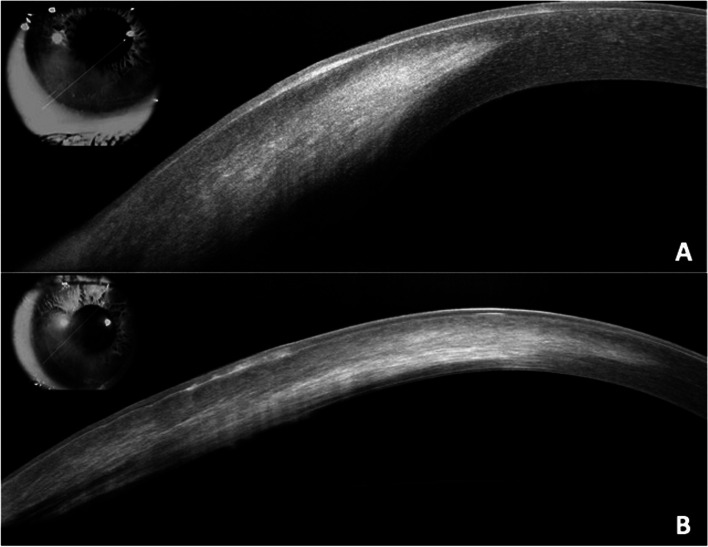
Anterior segment optical coherence tomography of the right (A) and left (B) corneas demonstrate deep stromal opacification

At presentation, the patient reported his only symptom to be bilaterally reduced vision, worse in his left eye. His best spectacle-corrected visual acuity was 20/80 in the right eye and 20/300 in the left eye. Intraocular pressures were 13 mmHg and 12 mmHg in the right and left eyes, respectively. No relative afferent pupillary defect was noted. Slit lamp evaluation demonstrated that his conjunctiva was white and quiet bilaterally. Examination of the right cornea revealed superficial and deep scarring with stromal vascularization inferotemporally extending approximately 2.5 mm from the limbus. The left cornea had a more advanced clinical picture, with inferonasal superficial and deep scarring and associated vascularization extending approximately 5.5 mm from the limbus into the visual axis (Fig. 1). The anterior chambers were deep and with no inflammation bilaterally. There was mild symmetric nuclear sclerosis that was consistent with the patient's age. Posterior segment examinations were unremarkable. Given the presence of significant corneal scarring, a rigid gas permeable contact lens over refraction was performed, which improved the visual acuity to 20/50 in the right eye and 20/80 in the left eye. The depth of corneal scarring was verified by anterior segment optical coherence tomography (Optovue Inc., Fremont, CA, USA) (Fig. 2).

The clinical picture of bilateral IK prompted laboratory investigation. Laboratory results were positive for Epstein-Barr virus viral capsid antigen IgG antibody, Epstein-Barr virus nuclear antigen IgG antibody, and herpes simplex virus 1 IgG antibody (Table 1). The remainder of the laboratory testing was negative, including evaluation for syphilis, Lyme disease, and tuberculosis.

**Table 1:** Laboratory results

Laboratory Test	Result (Reference Interval)
White blood cells	8.7 × 10^3^/uL (3.4–10.8)
Red blood cells	4.63 × 10^6^/uL (4.14–5.80)
Hemoglobin	14.3 g/dL (13.0–17.7)
Hematocrit	41.9% (37.5–51.0)
Platelets	297 × 10^3^/uL (150–450)
Neutrophils (absolute)	5.4 × 10^3^/uL (1.4–7.0)
Lymphocytes (absolute)	2.6 × 10^3^/uL (0.7–3.1)
Monocytes (absolute)	0.5 × 10^3^/uL (0.1–0.9)
Eosinophils (absolute)	0.1 × 10^3^/uL (0.0–0.4)
Basophils (absolute)	0.1 × 10^3^/uL (0.0–0.2)
*Immune and Infectious Markers*	
Angiogensin-converting enzyme	52 U/L (14–82)
Antinuclear Ab	Negative
EBV VCA, IgM Ab	< 36.0 U/ml (0.0–35.9)
EBV VCA, IgG Ab	**158.0 ↑** U/ml (0.0–17.9)
EBV NA, IgG Ab	** > 600.0 ↑** U/ml (0.0–17.9)
HSV 1, IgG Ab	**1.3 ↑** index (0.0–0.9)
HSV 2, IgG Ab	< 0.91 index (0.0–0.9)
HSV, IgM Ab	< 0.91 ratio (0.0–0.9)
Lyme total Ab	Negative
QuantiFERON-TB Gold Plus	Negative
Rapid plasma reagin	Non-reactive
Sedimentation rate	7 mm/hr (0–30)
Treponema pallidum Ab	Non-reactive
Varicella zoster, IgM Ab	< 0.91 index (0.0–0.9)
Varicella zoster, IgG Ab	2117 index (immune > 165)

*Ab* Antibody, *EBV* Epstein-Barr virus, *HSV* Herpes simpex virus, *NA* Nuclear antigen, *TB* Tuberculosis, *VCA* Viral capsid antigen

The patient was monitored on topical prednisolone acetate 1% four times daily in both eyes, and no improvement in vision or corneal opacification was noted over the ensuing 3 months. Topical steroids were subsequently tapered. Given his functional vision using rigid gas permeable contact lenses, the patient has opted to continue this management course and thus keratoplasty is not planned for the foreseeable future.

### Discussion and conclusions

The pathophysiology of COVID-19 involves systemic immune responses, with massive production of inflammatory mediators [2, 3]. The coronavirus enters host cells by binding its spike (S) protein to host ACE2 receptors, and the transmembrane serine protease 2 (TMPRSS2) facilitates viral fusion with the human cell [2, 3, 9]. Although the ACE2 receptor and TMPRSS2 are particularly expressed in type 2 alveolar epithelial cells, they have also been identified in several other tissues such as the conjunctiva, limbus, and cornea [2, 3].

The human body's immune reaction to SARS-CoV-2 infection involves innate and adaptive responses [9]. An intracellular cascade signal leads to the production of numerous proinflammatory cytokines such as tumour necrosis factor (TNF), interleukin 1 (IL-1) and 6 (IL-6), and interferons (IFNs) [2, 9]. IFNs typically protects the host from viral replication by inducing apoptosis of infected cells, although this cytokine can be supressed by SARS-CoV-2 proteins [9]. In the reported patient's case, an intense immunological response caused by viral particles may have led to severe injury to the corneal stroma, resulting in IK. This mechanism would be akin to that of stromal keratitis secondary to the herpes simplex virus, in which case herpes virus replication in the cornea triggers an immune signaling cascade and production of cytokines [10]. The resulting influx of inflammatory cells and antigen presenting cells result in both acute and chronic corneal inflammation and vascularization [10].

Approximately 11% of COVID-19 patients have ocular findings [1]. The most common ocular feature of this disease is viral conjunctivitis [1], and other anterior segment manifestations of COVID-19 include keratoconjunctivitis [11], episcleritis [4], and acute corneal graft rejection [5]. Additional ophthalmic manifestations include acute dacryoadenitis [12], cotton wool spots and retinal microhemorrhages [13], posterior scleritis [14], oculomotor nerve palsy [15], optic neuritis [16], Guillan-Barre syndrome [17], Miller Fisher syndrome [18], ophthalmic artery occlusion [19], and retinal vein occlusion [20]. As ophthalmic findings may precede systemic disease, knowledge of the ocular manifestations of COVID-19 is vital to permit early diagnosis and treatment.

IK involves chronic and nonulcerative inflammation of the corneal stroma with variable neovascularization, usually without epithelial or endothelial involvement [21]. The pathogenesis typically involves an immune-mediated response to foreign antigens, which are usually bacterial, viral, or parasitic [21]. At one institution in the United States, the most common identified causes of IK were herpes simplex virus and syphilis [22]. However, the vast majority of bilateral cases were either idiopathic or secondary to syphilis [22]. Other etiologies include Lyme disease, tuberculosis, Epstein-Barr virus, and acanthamoeba [21]. In our case, the possibility of keratitis due to Epstein-Barr virus or herpes simpex virus could not be definitively ruled out. Patients with severe COVID-19 infection have impaired immunity characterized by a reduction in the number of CD4 + and CD8 + T cells; reactivation or coinfection with other viruses have been well-documented among COVID-19 patients [23]. Although written clinical records of our patient revealed no prior corneal findings prior to COVID-19, we did not have photographic documentation of healthy corneas.

The management of IK typically involves topical inflammatory therapy and treatment of the underlying etiology, when identified [24]. ln our case, the patient was able to achieve functional vision with rigid gas permeable contact lenses. In conclusion, we present a rare case of bilateral IK after the onset of COVID-19 resulting in corneal scarring and decreased vision. We hope that this case highlights the importance of ocular evaluation in patients with COVID-19, as early management of IK may reduce ocular morbidity.

## Data Availability

Not applicable.

## Abbreviations

SARS-CoV-2: Severe acute respiratory syndrome coronavirus 2
ACE2: Angiotensin-converting enzyme 2
IK: Interstitial keratitis
PCR: Polymerase chain reaction
S: Spike protein
TMPRSS2: Transmembrane serine protease 2
TNF: Tumour necrosis factor
IL-1: Interleukin 1 and IL-6 6
IFNs: Interferons
